# Analysis of the trends and predictions of tuberculosis burden in China from 1990 to 2021 based on the GBD database

**DOI:** 10.3389/fpubh.2025.1626232

**Published:** 2025-09-01

**Authors:** Zhi-Qiang Lu, Shi-Cheng Feng, Min Feng, Jie Shen

**Affiliations:** ^1^Department of Radiotherapy, Zhongda Hospital, Southeast University, Nanjing, Jiangsu, China; ^2^Department of Radiology, Nanjing Chest Hospital, Affiliated Nanjing Brain Hospital, Nanjing Medical University, Nanjing, Jiangsu, China

**Keywords:** GBD database, tuberculosis, disease burden, predictive analysis, BAPC model

## Abstract

**Background:**

Tuberculosis (TB) is a major public health concern in China, exhibiting unique epidemiological traits and changing patterns. This study aims to assess the burden of TB in China from 1990 to 2021 and forecast the future.

**Methods:**

Data on TB burden indicators in China from 1990 to 2021 were collected from the Global Burden of Disease (GBD) database. The Joinpoint Regression (JPR) model was employed to assess trends in disease burden, with calculations of the annual percentage change (APC) and average annual percentage change (AAPC). The Auto-Regressive Integrated Moving Average (ARIMA) model and the Bayesian Age-Period-Cohort (BAPC) model were utilized to forecast trends in the age-standardized incidence rate (ASIR) and age-standardized mortality rate (ASMR) over the next 15 years.

**Results:**

From 1990 to 2021, the incidence, mortality, and disability-adjusted life years (DALYs) of TB in China showed a declining trend, decreasing by 47.17, 78.14, and 81.25%, respectively, while the absolute number of TB cases increased by 32.96%. In 2021, the ASIR, age-standardized prevalence rate (ASPR), ASMR, and age-standardized DALY rate (ASDR) of TB in China were 36.28 per 100,000 (95% CI: 32.63–40.47), 30,557.45 per 100,000 (95% CI: 27,692.69-33,531.31), 1.91 per 100,000 (95% CI: 1.51–2.51), and 76.22 per 100,000 (95% CI: 62.59–94.45), respectively, reflecting reductions of 66.60, 2.83, 90.72, and 89.53% from 1990 levels. The burden of TB exhibited disparities across gender and age groups, with older males experiencing a higher burden than older females, and children under 5 years old demonstrating the highest incidence rate among all age groups. The JPR regression model indicated a significant decline in ASIR (AAPC = −3.49; 95% CI: −3.49 to −3.37; *p* < 0.001), ASMR (AAPC = −7.42; 95% CI: −7.78 to −7.07; *p* < 0.001), and ASDR (AAPC = −7.01; 95% CI: −7.22 to −6.80; *p* < 0.001) from 1990 to 2021, whereas ASPR remained relatively stable (AAPC = −0.15; 95% CI: −0.37 to −0.006; *p* = 0.17). Predictions from both the ARIMA and BAPC models were consistent, suggesting a continued decline in ASIR and ASMR through 2036, with the burden remaining higher among males than females.

**Conclusion:**

From 1990 to 2021, TB incidence, mortality, and DALYs in China demonstrated an overall downward trend, with similar declines observed in both male and female populations. Projections indicate that ASIR and ASMR will continue to decline from 2022 to 2036. These findings provide valuable insights for the development of public health strategies aimed at reducing the TB burden in China.

## 1 Background

Tuberculosis (TB) is a highly infectious disease caused by *Mycobacterium tuberculosis*, primarily transmitted through airborne droplets (1). It is characterized by a prolonged incubation period, a lack of early symptoms, and high transmissibility (2). TB remains a major global health concern and is the second leading cause of death from infectious diseases. It poses a serious threat to public health and places a significant economic and social burden on countries worldwide. In 2021, the cost of TB diagnosis, treatment, and prevention services in low- and middle-income countries reached 5.4 billion USD (1). Despite these investments, model-based estimates in 27 countries indicate that TB morbidity and mortality may continue to rise (1).

China is among the high-burden countries for TB, ranking third among the 30 highest TB-burdened nations globally.

In 2022, China reported 500,000 new TB cases and 30,000 TB-related deaths, accounting for 7 and 2% of the global totals, respectively. Between 2015 and 2021, China’s TB control efforts achieved only an 8% reduction in incidence and a 15% reduction in mortality, falling significantly short of the WHO’s 2025 targets of a 50% reduction in incidence and a 70% reduction in mortality (3, 4). A growing concern is the country’s rapidly aging population, which may further hinder TB elimination efforts. The proportion of individuals aged 60 years and above in China increased from 13.26% in 2010 to 18.70% in 2021 and is projected to reach 28.0% (402 million people) by 2040. Notably, the prevalence of latent TB infection among the older population exceeds 30%, significantly higher than that observed in younger age groups (5). Moreover, studies indicate that older adults with TB exhibit a higher disease prevalence, lower treatment success rates, increased susceptibility to adverse drug reactions, and a greater risk of mortality (6), all of which present significant challenges to TB prevention and control in China. Therefore, a comprehensive assessment of the epidemiological characteristics of TB in China is essential to identify vulnerable populations and implement targeted intervention strategies (7).

Currently, the lack of high-quality data on TB incidence trends and the absence of a robust health monitoring system pose significant challenges to accurately assessing the TB burden and evaluating the effectiveness of public health policies and medical services (8). The GBD 2021 database, the largest international observational epidemiological research repository to date, includes comprehensive data since 1990, making it an ideal resource for studying long-term trends in TB incidence in China (9). Therefore, this study aims to analyze the TB burden in China from 1990 to 2021 using the latest GBD 2021 database. Additionally, we employ an ARIMA model and a BAPC model to predict future trends. Our findings will enhance the understanding of TB epidemiology and provide a theoretical foundation for the development of national public health policies.

## 2 Data and methods

### 2.1 Data sources

The GBD 2021 database comprises 75,459 data sources on non-fatal causes, including 36,916 records on morbidity, 22,236 on prevalence, and 45 on other epidemiological indicators (such as remission rates). It provides global estimates for morbidity, prevalence, disability-adjusted life years (DALYs), and healthy life expectancy, covering 288 causes of death and 88 risk factors (10). As a comprehensive study of global health burdens, GBD 2021 offers the most up-to-date information on the distribution and impact of diseases and injuries across different periods, age groups, sexes, locations, and socio-demographic groups. In this study, we utilized the GBD 2021 database to obtain data on TB cases, prevalence, mortality, and DALYs in China, including gender- and age-specific data. We analyzed age-standardized incidence rates (ASIR), ASPR, age-standardized mortality rates (ASMR), and ASDR across all age groups. Age groups were categorized into 20 five-year intervals, ranging from individuals younger than 5 years to those older than 95 years. The GBD study used anonymized, publicly available data and was approved by the Institutional Review Board of the University of Washington. Ethical approval and consent to participate are detailed on the following website: https://ghdx.healthdata.org/gbd-2021.

### 2.2 Joinpoint regression

The Joinpoint Regression (JPR) model, developed by the US National Cancer Institute (software version 5.0.2), was used in this study. Turning points in the temporal trend were identified by minimizing the sum of the squares of the residuals between estimated and actual values.^1^ The JPR model, a linear statistical model, was applied to describe the temporal trends in the TB burden, with the APC and AAPC calculated accordingly. The trends of ASIR, ASPR, ASMR, and ASDR of TB in China from 1990 to 2021 were analyzed and segmented into six sub-periods. For each sub-period, the APC and its 95% confidence interval (CI) were computed, while the AAPC was used to summarize the overall trend from 1990 to 2021. An APC > 0 with a lower bound of its 95% CI > 0 indicates an increasing trend in a specific period. Conversely, an APC < 0 with an upper bound of its 95% CI < 0 suggests a declining trend. If neither condition is met, the trend is considered stable (11). Statistical significance was set at *p* < 0.05.

### 2.3 ARIMA and BAPC models to predict TB disease burden trends

The ARIMA model was used to predict the prevalence and incidence of TB. The core principle of ARIMA involves differencing the time series to transform a non-stationary series into a stationary one for modeling. The ARIMA model consists of three key parameters: *p*, *d*, and *q*, where *p* represents the order of the autoregressive term, *d* denotes the degree of differencing, and *q* corresponds to the order of the moving average term. These parameters were determined using the autocorrelation function and partial autocorrelation function. The ‘forecast’ and ‘tseries’ packages were employed for forecasting and visualizing ARIMA models.

In addition to the ARIMA model, the BAPC model is also widely used in epidemiological forecasting. Compared with other forecasting methods, BAPC has higher coverage and accuracy (12). This statistical model is based on Bayesian statistical theory, which combines prior information about unknown parameters with sample information to estimate and infer the posterior distribution. The BAPC model is expressed as nij = log(λij) = *μ* + αi + βj + γk, where λij represents the number of cases, μ represents the intercept, and αi, βj, and γk represent the effects of age, period, and cohort, respectively. Standardised age structures and projected population data from the GBD come from World Population Prospects 2022.^2^ The TB ASIR and ASMR burden of disease for 2022–2036 was projected using the ‘BAPC’ and ‘INLA’ packages (13, 14), and the BAPC model was visualized. All data analyses were performed using the open-source software R (version 4.2.1).

## 3 Results

### 3.1 Changes in the burden of TB disease in China from 1990 to 2021

From 1990 to 2021, the incidence numbers of TB cases in China declined from 1.1678 million in 1990 to 617,700 in 2021, representing a 47.17% reduction. Meanwhile, the ASIR decreased from 109.01 per 100,000 population (95% CI: 94.81–124.61) in 1990 to 36.28 per 100,000 (95% CI: 32.63–40.47) in 2021, marking a 66.60% decline. However, the number of people affected increased from approximately 370 million in 1990 to 492 million in 2021, a 32.96% rise. The ASPR slightly decreased from 31,445.76 per 100,000 in 1990 (95% CI: 27,902.18-35,142.48) to 30,557.45 per 100,000 in 2021 (95% CI: 27,692.69-33,531.31), a marginal reduction of 2.83%. The relatively stable number of TB cases despite a decline in incidence may be primarily attributed to population growth. Regarding mortality, the number of TB-related deaths fell from 171,100 in 1990 to 37,300 in 2021, a substantial 78.14% reduction, suggesting a decline in TB lethality. The ASMR sharply declined from 20.10 per 100,000 in 1990 (95% CI: 16.68–23.84) to 1.91 per 100,000 in 2021 (95% CI: 1.51–2.51), reflecting a 90.72% decrease and a significant improvement in TB survival outcomes.

In terms of health burden, DALYs due to TB dropped from approximately 73.26 million in 1990 to 13.76 million in 2021, an 81.25% reduction, indicating a significant decrease in years of healthy life lost to TB. The ASDR declined from 719.42 per 100,000 in 1990 (95% CI: 610.63–837.38) to 76.22 per 100,000 in 2021 (95% CI: 62.59–94.45), representing an 89.53% decrease. Overall, between 1990 and 2021, China’s ASIR, ASPR, ASMR, and ASDR for TB exhibited varying degrees of decline, highlighting the positive impact of TB control efforts. See Table 1 for detailed statistics.

**Table 1:** The disease burden of TB in China from 1990 to 2021.

Measure	1990		2021	
	All-ages cases (95% CI)	Age-standardized rates per 100,000 people(95% CI)	All-ages cases (95% CI)	A Age-standardized rates per 100,000 people(95% CI)
Incidence	1,167,808 (1001441–1,359,621)	109.01 (94.81–124.61)	617,726 (549548–688,348)	36.28 (32.63–40.47)
Prevalence	369,779,785(324342084–420,948,319)	31445.76 (27902.18–35142.48)	491,548,380 (445855387–539,371,580)	30557.45 (27692.69–33531.31)
Deaths	171,091 (141634–204,908)	20.10 (16.68–23.84)	37,332 (29309–49,368)	1.907 (1.51–2.51)
DALYs	7,325,730 (6221452–8,528,411)	719.42 (610.63–837.38)	1,375,510 (1120820–1,723,072)	76.22 (62.59–94.45)

TB, tuberculosis; CI, confidence interval; DALYs, the disability-adjusted life years.

### 3.2 Analysis of the gender and year distribution of TB patients in China

**Figure 1:**
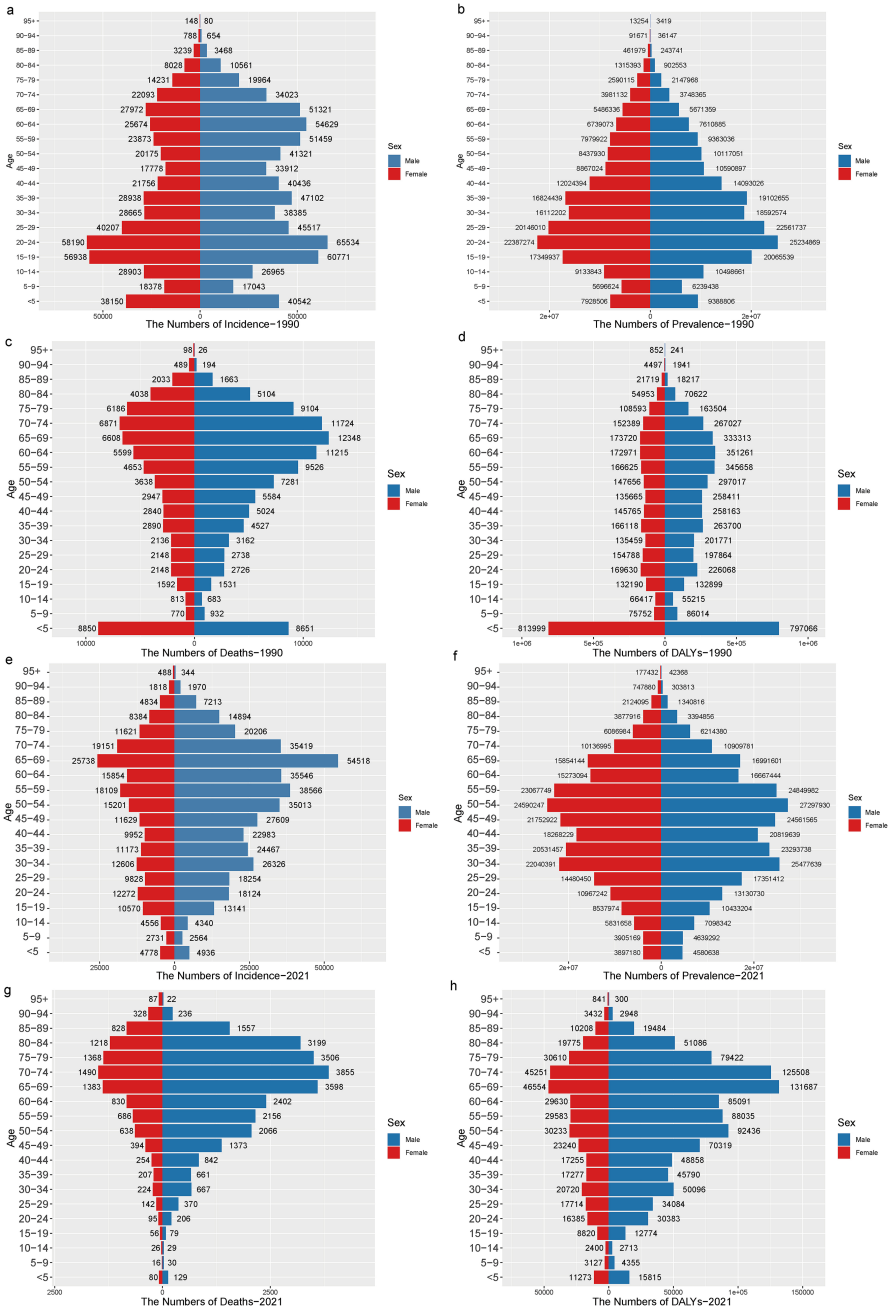
The incidence, prevalence, mortality, and DALYs of TB in males and females across different age groups in China from 1990 to 2021. **(a-d)** The incidence, prevalence, mortality, and DALYs of TB in 1990 were respectively; **(e-h)** The incidence, prevalence, mortality, and DALYs of TB in 2021 were respectively.

In 1990, the number of TB cases, the number of people affected, the number of deaths, and DALYs were higher for children under 5 years of age compared to other age groups. The peak age for TB incidence in China was 15–24 years. After the age of 25, the incidence rate generally declined with age. A slight increase in cases was observed between the ages of 50 and 70, with the number of male cases being higher than that of females. The number of people affected showed an increasing trend after the age of 5, reaching a peak in the 20–24 age group, before declining with increasing age. The number of deaths from TB increased with age, with the highest number of deaths concentrated in the age groups under 5 years and 70–74 years, gradually decreasing after reaching this peak. DALYs in the population older than 5 years showed little variation with age, and in all age groups, except the 10–14 age group, the number of DALYs for males was higher than for females (Figures 1a–d).

By 2021, the peak age for TB incidence in China had shifted to 65–69 years, with 54,518 cases in men, approximately twice as many as in women. The highest TB incidence remained concentrated in the 30–34 and 50–54 age groups. The number of deaths continued to rise with age, with the highest number occurring in the 70–74 age group, predominantly in older males (>65 years). Regarding the reduction of TB burden, the DALY results mirrored the trends seen in mortality, peaking in the 65–74 age group, with males consistently experiencing a higher number of DALYs than older females (>65 years). Progress in reducing the burden of TB varied by age group, with the most significant reductions observed in children (<5 years old) and the older population. Throughout the period from 1990 to 2021, the number of cases, the number of people affected, the number of deaths, and the DALYs for both males and females all declined to varying degrees, with males consistently showing higher numbers than females (Figures 1e–h).

**Figure 2:**
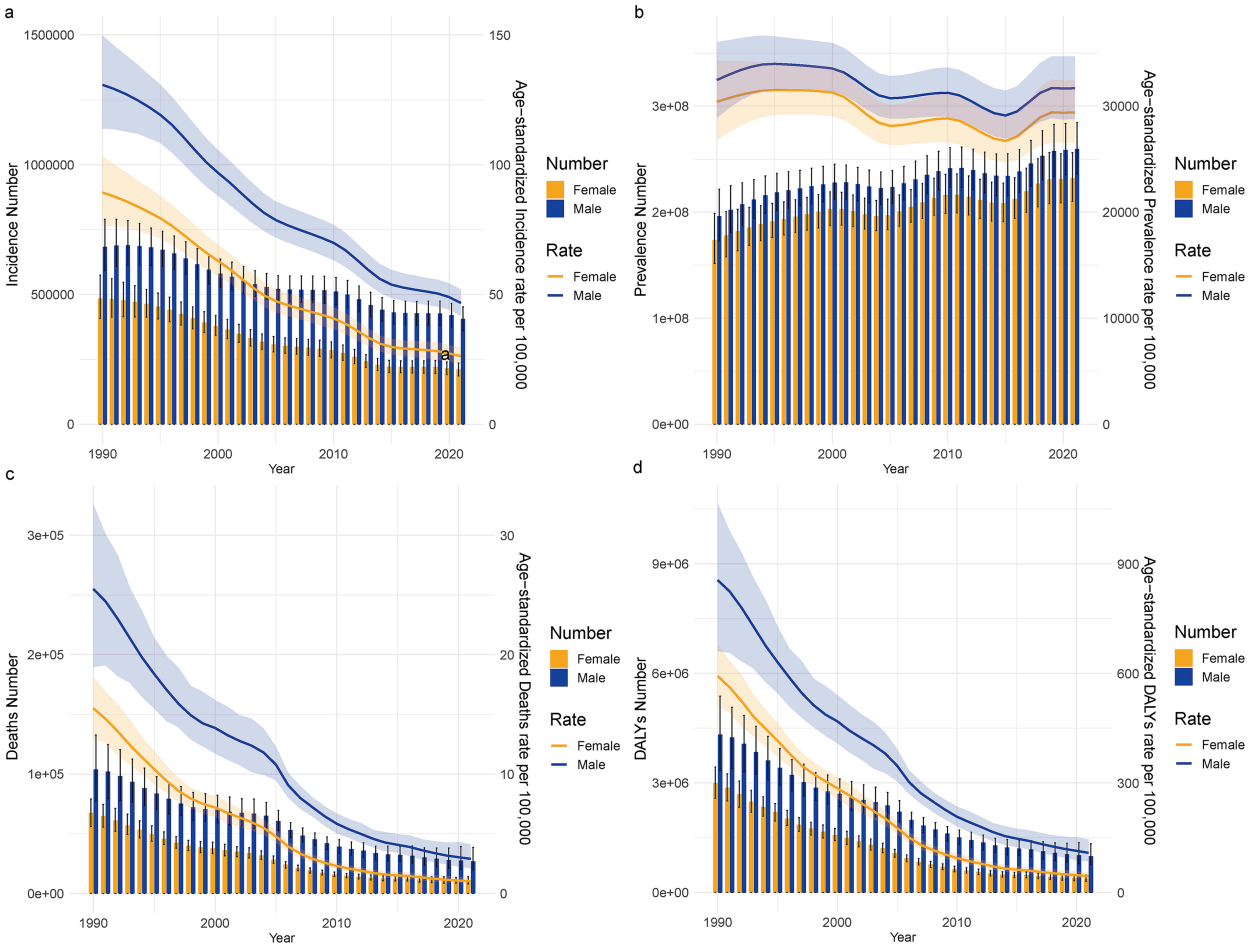
The incidence, prevalence, mortality, and disability-adjusted life years (DALYs) for male and female TB in China from 1990 to 2021, along with their standardized rates. **(a)** Number of patients and ASIR; **(b)** Number of patients and ASPR; **(c)** Death cases and ASMR; **(d)** DALYs count and ASDR.

A comparison of the disease burden and age-standardized rates of TB in males and females of all ages in China from 1990 to 2021 shows that the ASIR peaked in 1990, with a substantial difference between men and women. Over time, the ASIR showed a consistent decline, and the gap between men and women gradually narrowed. From 1990 to 2021, ASPR for TB in both males and females followed a wave-like pattern, with slight increases around 2010 and 2020, and declines around 2005 and 2015. The ASMR and ASDR exhibited similar trends, with both rates showing a year-on-year decline. However, the ASMR and ASDR for males remained consistently higher than for females, and the difference between the two tended to narrow over time (Figure 2).

### 3.3 JPR model analysis of TB trends

**Figure 3:**
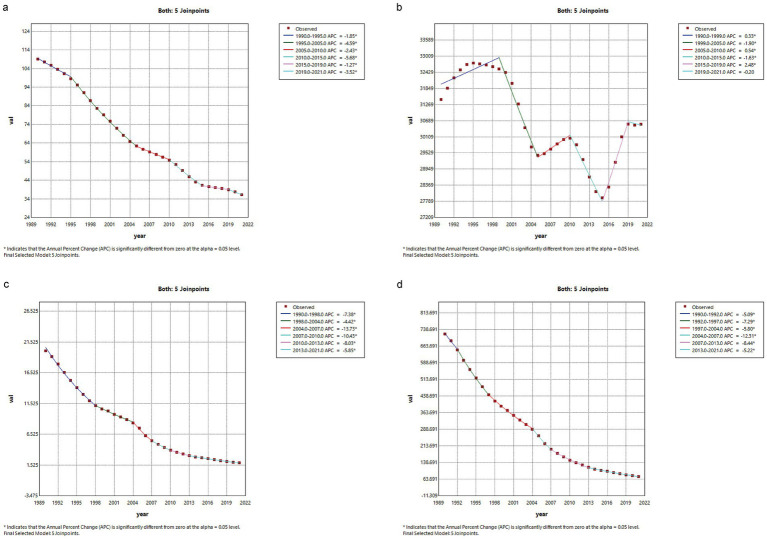
JPR Model Analysis of the Overall Population Burden of TB Disease in China from 1990 to 2021. **(a)** ASIR **(b)** ASPR **(c)** ASMR **(d)** ADIR.

The results of the JPR model indicate that from 1990 to 2021, the ASIR, ASMR, and ASDR of TB in the general population of China all exhibited a year-on-year decrease. Notably, the ASIR showed the greatest decline between 2007 and 2010, with an APC of −10.43% (*p* < 0.05), and the AAPC was −3.49% (95% CI: −3.61 to −3.37), corresponding to an average annual decrease of 3.49% (*p* < 0.001). In contrast, the ASPR displayed a wave-like trend, showing upward trends from 1990 to 1999, from 2005 to 2010, and from 2015 to 2019, with APCs of 0.33, 0.54, and 2.48% (*p* < 0.05), respectively. The largest increase occurred between 2015 and 2019. A significant decline in ASPR occurred between 1999 and 2005, with an APC of −1.90% (*p* < 0.05), followed by a slight change between 2019 and 2021, with an APC of 0.20%. The AAPC for ASPR from 1990 to 2021 was −0.15% (95% CI: −0.37 to 0.06), with a t-value of −1.38 and a *p*-value of 0.17. This suggests a slight decrease in ASPR during this period, although the decrease was not statistically significant, possibly influenced by the impact of the COVID-19 pandemic or other factors (Figure 3).

From 1990 to 2021, the ASMR showed a significant decrease, with an AAPC of −7.42% (95% CI: −7.78 to −7.07), reflecting an average annual reduction of 7.42% (*p* < 0.001). The most notable decrease in ASMR occurred between 2004 and 2007, with an APC of −13.41% (*p* < 0.05). Similarly, the ASDR showed a downward trend, with a significant decrease between 2010 and 2015, where the APC was −5.68% (*p* < 0.05). The AAPC for ASDR from 1990 to 2021 was −7.01% (95% CI: −7.22 to −6.80; *p* < 0.001), indicating a 7.01% average annual reduction in ASDR, underscoring significant progress in reducing TB-related health losses in China (Table 2).

**Table 2:** AAPC of TB ASIR, ASPR, ASMR, and ASDR in China from 1990 to 2021.

Measure	Period	AAPC	95% CI		*t*-value	*p*-value
ASIR	1990–2021	−3.49	−3.61	−3.37	−56.64	<0.001
ASPR	1990–2021	−0.15	−0.37	0.06	−1.38	0.17
ASMR	1990–2021	−7.42	−7.78	−7.07	−39.53	<0.001
ASDR	1990–2021	−7.01	−7.22	−6.80	−63.10	<0.001

TB, tuberculosis; CI, confidence interval; AAPC, the average annual percentage change; ASIR, the age-standardized incidence rate (ASIR); ASMR, the age-standardized mortality rate; ASPR, the age-standardized prevalence rate; ASDR, the age-standardized DALY rate.

### 3.4 ARIMA and BAPC models predict the trend of TB disease burden in China

**Figure 4:**
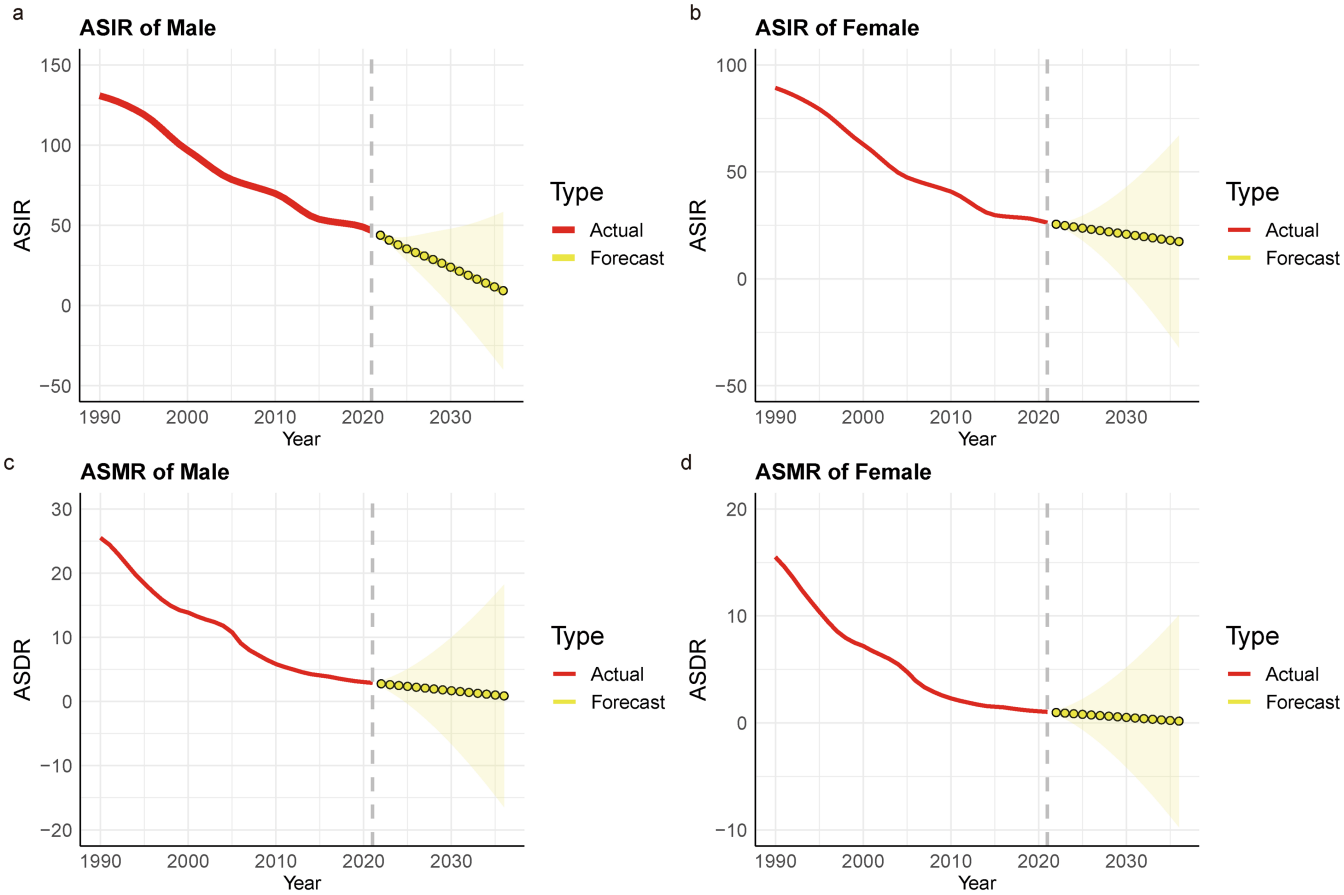
Prediction of TB ASIR and ASMR in China based on the ARIMA model. **(a)** Male -ASIR; **(b)** Female -ASIR; **(c)** Male -ASMR; **(d)** Female –ASMR.

**Figure 5:**
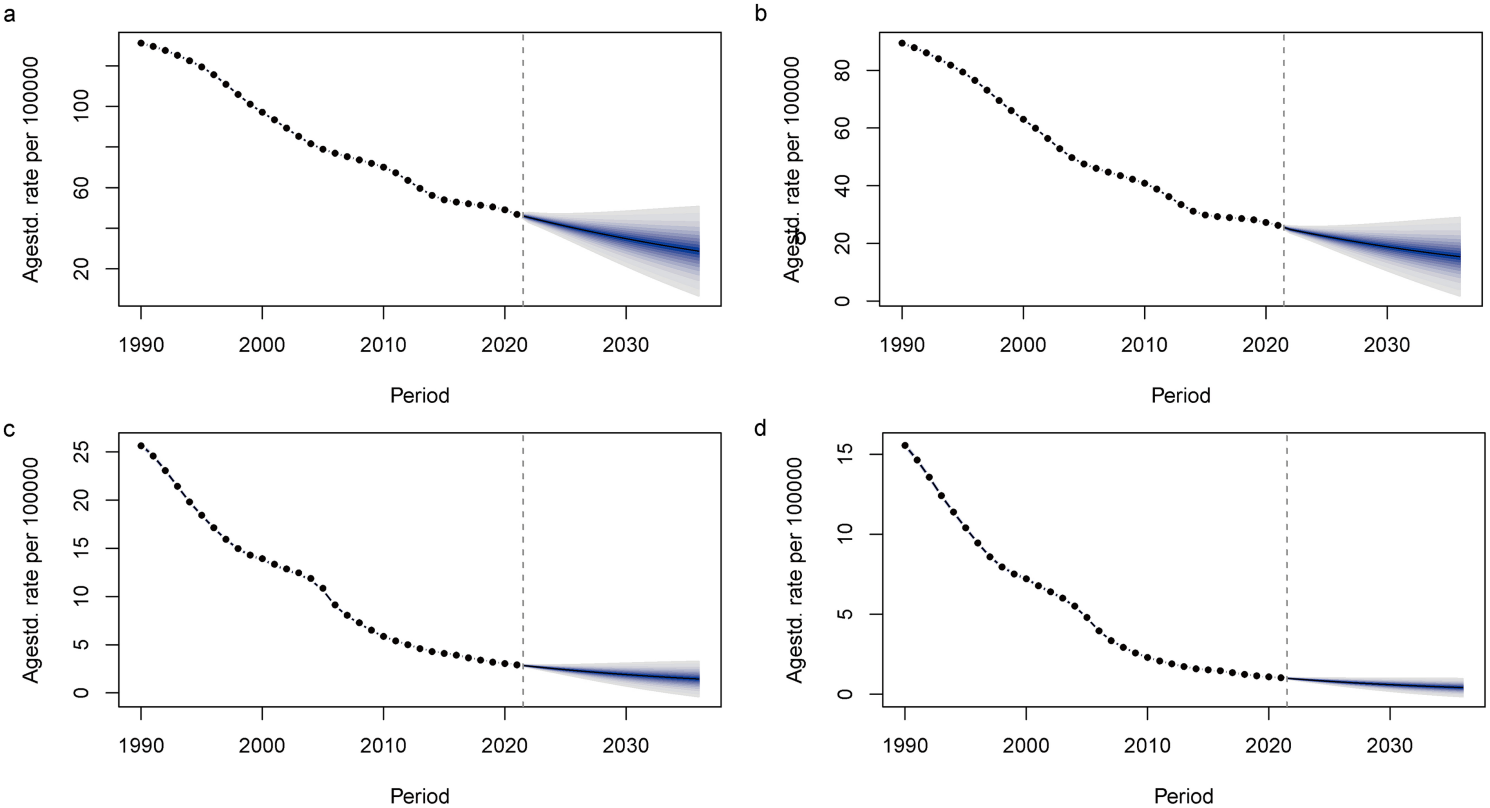
The Trends of TB ASIR and ASMR Predicted by the BAPC Model in China. **(a)** Male -ASIR; **(b)** Female -ASIR; **(c)** Male -ASMR; **(d)** Female -ASMR.

According to the ARIMA model, the ASIR and ASMR of Chinese men and women are predicted to decline to varying degrees from 1990 to 2036, and the disease burden of men will be greater than that of women. Among them, the ASIR of men will decline from 131.11 per 100,000 in 2021 to 28.69 per 100,000 in 2036, from 26.26/100,000 in 2021 to 1.54/100,000 in 2036; among them, ASMR, male from 2.90/100,000 in 2021 to 0.87/100,000 in 2036, female from 1.03/100,000 in 2021 to 0.17/100,000 in 2036 (Figure 4). The BAPC model indicates that between 1990 and 2021, both the ASIR and ASMR for TB patients, in both men and women, exhibited a year-on-year decrease, with similar trends observed for both sexes. Furthermore, the BAPC model predicts a similar trend to the ARIMA model, forecasting a continued year-on-year decline in both the ASIR and ASMR for men and women from 2022 to 2036, with rates tending toward 0 per 100,000 (Figure 5).

## 4 Discussion

This study analyzed the current prevalence of TB and the changing trends in disease burden in China from 1990 to 2021, based on the GBD 2021 database. The results indicate that as of 2021, China’s ASIR, ASPR, ASMR, and ASDR for TB all showed a year-on-year decline, with decreases of 66.60, 2.83, 90.72, and 89.53%, respectively, compared to 1990. From 1990 to 2021, there were significant gender differences in the TB burden in China. In most age groups, the trends in disease burden for males and females were consistent with the overall trend, with the ASIR, ASPR, ASR, and ASDR for males being higher than for females. This finding aligns with the WHO report, which indicates that the proportion of male TB cases is higher (1). Li et al. analyzed the age-specific reported incidence rate from the Chinese tuberculosis information management system (2008–2018) and found that the reported rate of pulmonary tuberculosis in males was more than twice that of females (15). Differences in TB incidence rates between sexes are evident, with controlling TB incidence rates being less effective in males than in females (8). Men in birth cohorts from countries with below-average population indices are at higher risk of TB progression (8). Studies have shown that in the male TB population, adverse factors such as smoking, alcohol abuse, diabetes, chronic obstructive pulmonary disease and chronic work stress are more closely associated with the disease. HIV-TB co-infection is a significant public health challenge as the two illnesses mutually aggravate one another, resulting in heightened morbidity and mortality rates (16). These factors not only significantly increase the risk of TB but also negatively affect treatment outcomes (8). Therefore, a gender-specific approach is essential in TB prevention and treatment strategies.

Zhang Ting and colleagues have shown that the global burden of tuberculosis is primarily concentrated in individuals aged 40–60, although the pathogenesis remains unclear (17). The susceptibility of the older population to multidrug-resistant TB increases due to prolonged use of anti-TB drugs and a gradual decrease in immunity with age (18). Teng Rencong et al. analyzed the Chinese TB information management system and found that, based on the registration of older population TB patients from 2015 to 2021, the reported incidence of TB in individuals aged ≥65 years showed a downward trend (19). However, the proportion of older population individuals among all TB patients has increased year by year, suggesting that the older population continue to be a key population in China’s TB prevention and control efforts. To achieve the TB control goals set by the WHO, TB prevention and control efforts in the older population in China should prioritize early case detection, improve the quality of TB treatment, and provide long-term treatment post-TB (20). The prevalence of TB is common among the older population, and the reporting rate gradually increases with age. Most cases of TB in the older population are related to the reactivation of the focus that was still dormant (6). Enhancing the primary health care system, particularly in rural regions, is advised to augment access to health care and the surveillance of the medical state of the older population (21).

Dong et al. used an age-period-cohort model to analyze the data and found that the risk of tuberculosis among young people (aged 20–24) and the older population (aged 70–74) was similar (22). This study indicates that the downward trend in the TB burden in China is not uniform across different age groups. From 1990 to 2021, the number of TB cases in children under 5 years old in China decreased significantly, but this age group remains the peak for TB incidence, a finding consistent with previous studies in the United States (23). Children under 15 years of age are also a vulnerable group for tuberculosis due to their weaker immune systems, and there is a significant issue of underreporting of TB cases in this demographic (24). Between 2015 and 2020, the incidence of TB in children under 15 years of age decreased by 16%, while the mortality rate fell by 34% (3). The WHO’s Global Tuberculosis Report 2023 indicates that children accounted for 2% of notified TB cases in China in 2022, which is much lower than the global estimated proportion of 12%. And the Centers for Disease Control and Prevention indicates that China has attained considerable clinical and societal advantages, swiftly decreasing the incidence of TB (21). China has made substantial progress in reducing the burden of childhood TB, with varying degrees of decline in both the number of cases and affected individuals, primarily due to increased BCG vaccination coverage and enhanced TB surveillance among children.

The COVID-19 pandemic has hindered the timely diagnosis, reporting, and effective treatment of TB, resulting in an increase in TB mortality and reversing the progress made by the WHO in reducing the TB burden before 2019 (25). Worldwide, countries are continuously optimizing their TB control programs to meet the 2035 targets. In China, especially for the older population, non-pharmaceutical interventions, traffic restrictions, and the reallocation of medical resources during the COVID-19 pandemic led to a decrease in TB consultations and disruption of medical services (26). However, analysis using the JPR regression model shows that from 1990 to 2021, China’s ASIR, ASMR, and ASDR for TB generally decreased, while the ASPR remained relatively stable. This stability may be attributed to the increase in population size, which resulted in a non-significant decrease in the prevalence rate. According to predictions from the ARIMA and BAPC models, the ASIR and ASMR for TB are expected to continue declining over the next 15 years for both men and women. The BAPC model provides a more optimistic outlook, predicting that the number of TB cases and deaths will approach zero by 2036. Despite an aging population and increasing life expectancy, both models offer optimistic predictions, likely reflecting ongoing improvements in TB treatment and China’s healthcare system. Meanwhile, Li et al. used a gray model [GM(1, 1)] to predict that the number of TB deaths in China will show a downward trend by 2027, which is consistent with the findings of this study (27). The Long Short-Term Memory model exhibits superior accuracy compared to the ARIMA and GM (1, 1) models. Its predictive outcomes can serve as a valuable tool for tuberculosis control strategies in China (28). Recent study indicates that regional disparities in the tuberculosis burden among China’s provinces are associated with societal factors and variations in prevention and control programs (29). The uncertainty range in the predictive model varies, considering alterations in population size, enhancements in TB control efficacy, and numerous influencing factors such as HIV infection, smoking, and diabetes. The interrelated and intricate aspects render precise forecasting of TB developments difficult. To continue reducing the disease burden of TB in China, there is a need for improved prevention and control strategies, stronger policy support, and increased financial investment (30).

In the fight against TB, china has actively promoted the disease control process and has achieved full coverage of the Directly observed treatment and short course chemotherapy (DOTS) strategy around 2004 (31). Chinese primary healthcare (PHC) sectors implement the TB Control Program to improve active case finding, referral, treatment adherence, and health education (32). To accelerate the progress toward ending TB, the Chinese government implemented the End TB Action Plan (2019–2022), Among the Plan, health promotion was conducted to improve the awareness of TB knowledge among Chinese people (33).

This study has several limitations in evaluating the trend of TB disease burden in China. First, the GBD database may introduce certain biases in data collection and model construction, and it faces issues such as incomplete data. Second, the disability weights used in the calculation of DALYs are based on survey data that may not be fully applicable to the historical context of China, and therefore, they may not accurately reflect the disease burden of TB patients in China over time. Third, our prediction of future TB burden is based on current trends and patterns, without considering the potential future impact of changes in risk factors, advances in treatment technology, or adjustments in healthcare policies. Fourth, this study primarily relies on aggregate data when assessing TB incidence and mortality, and does not fully incorporate individual-level factors that may influence the disease burden, such as BCG vaccination status or the prevalence of anti-tuberculosis treatment.

## 5 Conclusion

In summary, TB continues to impose a significant disease burden in China. However, due to the country’s emphasis on TB control and the ongoing improvements in the diagnosis and treatment of infectious diseases, the incidence and mortality rates of TB have been effectively controlled. The prediction models also suggest that the overall TB disease burden in China is expected to improve over the next 15 years.

## Data Availability

The original contributions presented in the study are included in the article/supplementary material, further inquiries can be directed to the corresponding author.

## Footnotes

^1^ https://surveillance.cancer.gov/joinpoint/download

^2^ https://population.un.org/wpp/downloads?folder=Standard%20Projections&group=CSV%20format
